# *Scutellaria baicalensis* Alleviates Cantharidin-Induced Rat Hemorrhagic Cystitis through Inhibition of Cyclooxygenase-2 Overexpression

**DOI:** 10.3390/molecules17066277

**Published:** 2012-05-25

**Authors:** Steven Kuan-Hua Huan, Kun-Teng Wang, Shauh-Der Yeh, Chia-Jung Lee, Li-Chun Lin, Der-Zen Liu, Ching-Chiung Wang

**Affiliations:** 1 Division of Urology, Department of Surgery, Chi Mei Medical Center, No.21, Taikang, Liuying Dist., Tainan City 73657, Taiwan; Email: skhsteven@yahoo.com.tw; 2 Graduate Institute of Clinical Medical Research, Taipei Medical University, 250 Wu-Hsing Street, Taipei City 11031, Taiwan; Email: d8602003@tmu.edu.tw; 3 School of Pharmacy, College of Pharmacy, Taipei Medical University, 250 Wu-Hsing Street, Taipei City 11031, Taiwan; Email: b8706014@tmu.edu.tw (K.-T.W.); snoopy0615@hotmail.com (C.-J.L.); m303092003@tmu.edu.tw (L.-C.L.); 4 Section of Biologics & Advanced Therapeutic Product Analysis, Division of Research and Analysis, Food and Drug Administration, No.161-2, Kunyang St, Nangang District, Taipei City 11561, Taiwan; 5 Graduate Institute of Biomedical Materials, Taipei Medical University, 250 Wu-Hsing Street, Taipei City 11031, Taiwan; Email: tonyliu@tmu.edu.tw

**Keywords:** mylabris, cantharidin, *Scutellaria baicalensis*, hemorrhagic cystitis, cyclooxygenase-2, hematuria, c-Fos

## Abstract

Cantharidin, an active component in mylabris, is used in traditional Chinese medicine (TCM) to treat scabies and hepatoma, but accompanied by hemorrhagic cystitis. Evidence shows that cantharidin induces human bladder carcinoma cell death through COX-2 overexpression *in vitro*. In TCM, *Scutellaria baicalensis* is usually used to cure mylabris-induced hematuria. This work was undertaken to determine the mechanisms of cantharidin-induced rat hemorrhagic cystitis and explore the uroprotective effect of *S. baicalensis. In vitro* results showed cantharidin could induce cytotoxicity through prostaglandin (PG)E_2_ overproduction of T24 cells. Boiling-water extract of *S. baicalensis* (SB-WE) could significantly inhibit PGE_2_ production and COX-2 expression in lipo-polysaccharide-induced RAW 264.7 cells, indicating obvious anti-inflammatory abilities. *In vivo* results indicated that cantharidin caused rat hemorrhagic cystitis with hematuria via c-Fos and COX-2 overexpression. SB-WE was given orally to cantharidin-treated rats, whereby hematuria level, elevated PGE_2_ and COX-2 protein overexpression were significantly and dose-dependently inhibited by SB-WE. The anti-inflammatory components of SB-WE are baicalin and wogonin, whose contents were 200.95 ± 2.00 and 31.93 ± 0.26 μg/mg, respectively. In conclusion, cantharidin induces rat cystitis through c-Fos and COX-2 over-expression and *S. baicalensis* can prevent the resulting hematuria because of its anti-inflammatory effects.

## 1. Introduction

Cantharidin is a major component of mylabris (*Mylabris phalerata* Pallas), commonly known as Spanish Fly. In Europe and India, mylabris is used as an aphrodisiac. In traditional Chinese medicine (TCM), people use it to treat scabies and hepatoma [1,2]. However, people who take an overdose of mylabris will be poisoned by cantharidin. According to clinical case reports, cantharidin poisoning results in burning of the mouth, hematemesis, hepatomegaly, irritation of the genitourinary tract, hematuria, and dysuria [1,3,4]. Moreover, in a previous study we found that cantharidin induced secondary necrosis and cyclooxygenase (COX)-2 overexpression in bladder epithelial cells [5]. 

In TCM, doctors use *Scutellaria baicalensis* Georgi (Labitae) to cure mylabris-induced hematuria [6]. *S. baicalensis* is recorded for its traditional heat-clearing and damp-drying properties and used to treat respiratory tract infections, vigorous fever, diarrhea, spontaneous external bleeding, jaundice, and hepatitis. In addition, *S. baicalensis* also plays a therapeutic role in anti-pyretic and detoxification treatments [7,8,9,10,11,12]. In terms of its chemical components, *S. baicalensis* is rich in flavonoids, including chrysin, baicalein, baicalin, 7-methylbaicalein, norwogonin, oroxylin A, scutellarein, wogonin, and wogonoside [13,14]. Among the various components in *S. baicalensis*, baicalin and wogonin are the two major ones and are reported to have anti-inflammatory properties. In Lin’s study, baicalin showed significant anti-inflammatory effects on LPS-induced raw paw edema. Wogonin also displayed PGE_2_ down-regulative effects in interleukin-1 beta or tumor necrosis factor-alpha-induced NIH/3T3 cell lines [15,16,17]. Furthermore, baicalin and wogonin were well-documented to promote urination in anesthetized dogs, rabbits, and mice [18]. 

Nowadays, few study discuss rat hemorrhagic cystitis. Researchers usually use cyclophosphamide and ifosfamide, which are DNA-alkylating agents used in cancer therapy, as animal cystitis inducers. Metabolites of cyclophosphamide and ifosfamide can induce urinary bladder irritation and hematuria through COX-2 and inducible nitric oxide synthase (iNOS) overexpression [19,20]. Based on the above reasons, we assumed that cantharidin might cause bladder inflammation and then induce hematuria, via a mechanism which possibly the same as that for cyclophosphamide and ifosfamide. 

In this study, we tried to establish a cantharidin-induced rat hematuria model by directly injecting cantharidin into the bladder of female Wistar rats and discuss the mechanism of its irritation. The boiling-water extract of *S. baicalensis* (SB-WE) was also used to treat cantharidin-induced rat hematuria and we explored its uroprotective mechanism. *In vitro* nitric oxide (NO) and prostaglandin (PG)E_2_ inhibition abilities and *in vivo* immunohistochemistry, hematuria level and COX-2 expression of rat bladder were used to evaluate the uroprotection of SB-WE. Besides, the quality of the SB-WE was monitored by the levels of two substance markers, baicalin and wogonin, with a high-performance liquid chromatographic (HPLC) method.

## 2. Results

### 2.1. Cantharidin Induced T24 Cell Death

**Figure 1:**
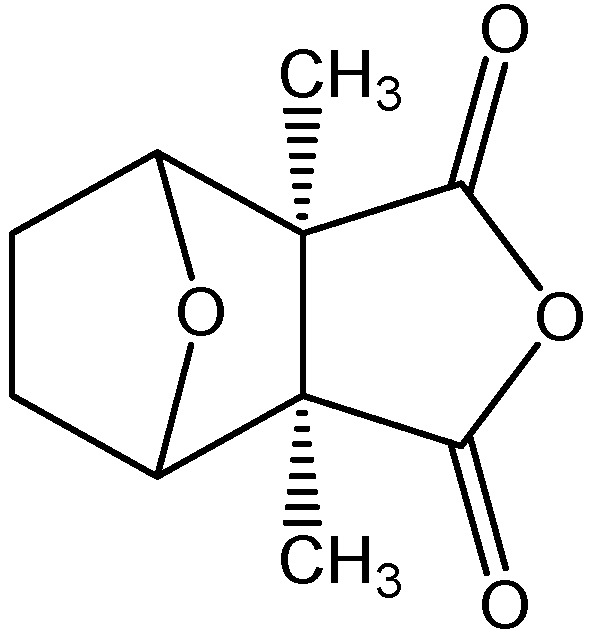
Structure of cantharidin.

The structure of cantharidin (2,3-dimethyl-7-oxabicyclo[2.2.1]heptane-2,3-dicarboxylic acid anhydride, C_10_H_12_O_4_, m.w. 196.2) is displayed in Figure 1. It is an odorless and colorless powder.

**Figure 2:**
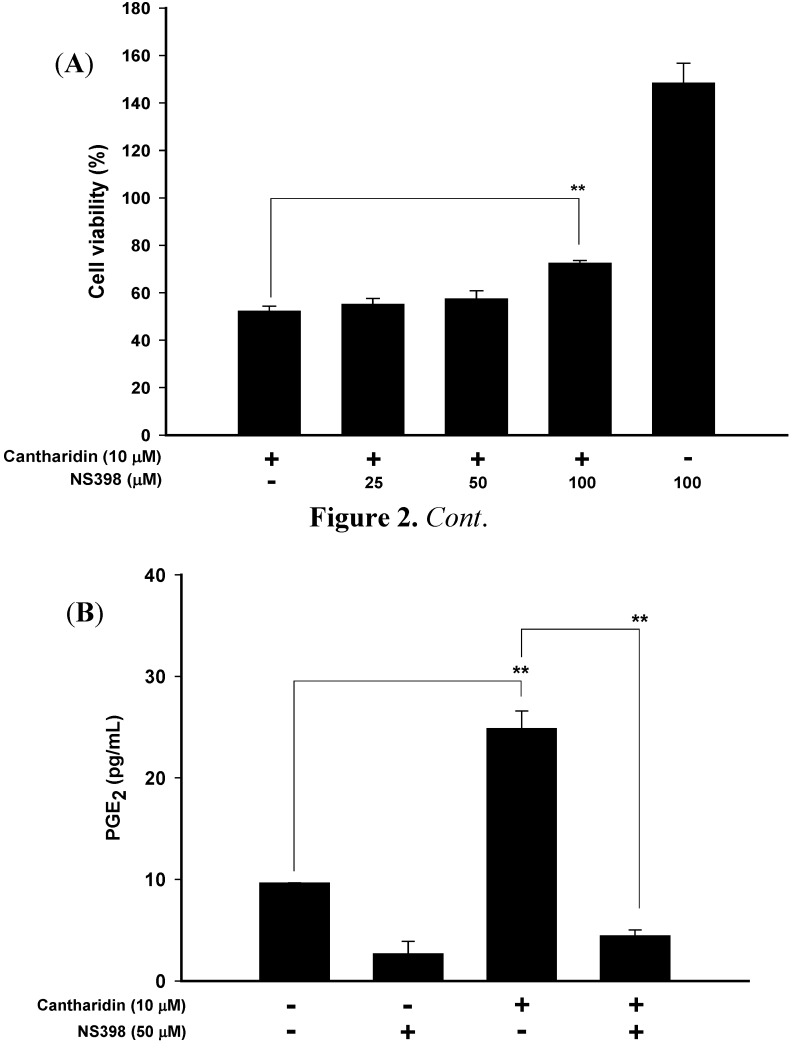
NS398 significantly inhibited cantharidin-induced (**A**) cytotoxicity and (**B**) PGE_2_ production in T24 cells. NS398: A selective COX-2 inhibitor. ** *p* < 0.001. *n* = 3.

Figure 2A shows that cantharidin significantly induced T24 cell necrosis at 10 μM. To explore whether cantharidin resulted in cell death through COX-2 expression and PGE_2_ production, NS398, a cyclooxygenase-2-specific inhibitor, was used.

As shown in Figure 2A/2B, NS398 significantly ameliorated the cytotoxicity at 100 μM and decreased PGE_2_ production in cantharidin-treated T24 cells at 50 μM. These results suggest that the cantharidin-induced cell death could be attenuated through treatment with anti-inflammatory agents. 

### 2.2. Cantharidin Induced Rat Hemorrhagic Cystitis Through Initiating Urinary Bladder Inflammation

**Figure 3:**
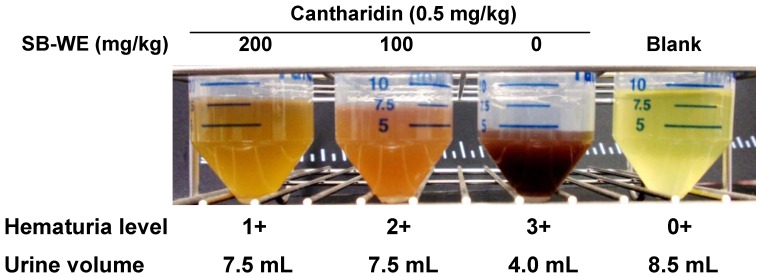
The boiling-water extract of *S. baicalensis* (SB-WE) dose-dependently alleviated cantharidin-induced hematuria and dysuria in Wistar rats.

In an *in vivo* assay, cantharidin (0.5 mg/kg) was directly injected into the bladder of female rats, where it induced severe hematuria and dysuria after 16 h (Figure 3). 

**Figure 4:**
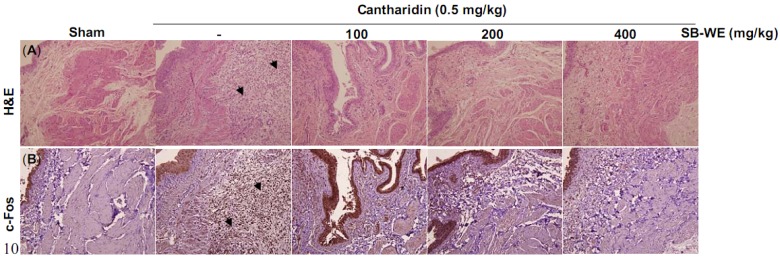
(**A**) Hematoxylin and eosin (H&E) and (**B**)immunohistochemical stainingof c-Fos to elucidate the uroprotective effects of the boiling-water extract of *S. baicalensis* (SB-WE) on cantharidin-induced rat cystitis. The solid black triangle pointed out the neutrophil infiltrations and significant c-Fos signal.

The pathology examination showed that cantharidin induced colliquative necrosis of the urinary bladder epithelium and induction of neutrophil infiltration in cantharidin-treated Wistar rats (Figure 4A). Besides, the immunohistochemistry results showed that c-Fos, an inflammation-related transcription factor, was significantly increased in rat bladder after cantharidin injection (Figure 4B). The urinary bladders were homogenized and protein extracted for the Western blot analysis. 

**Figure 5:**
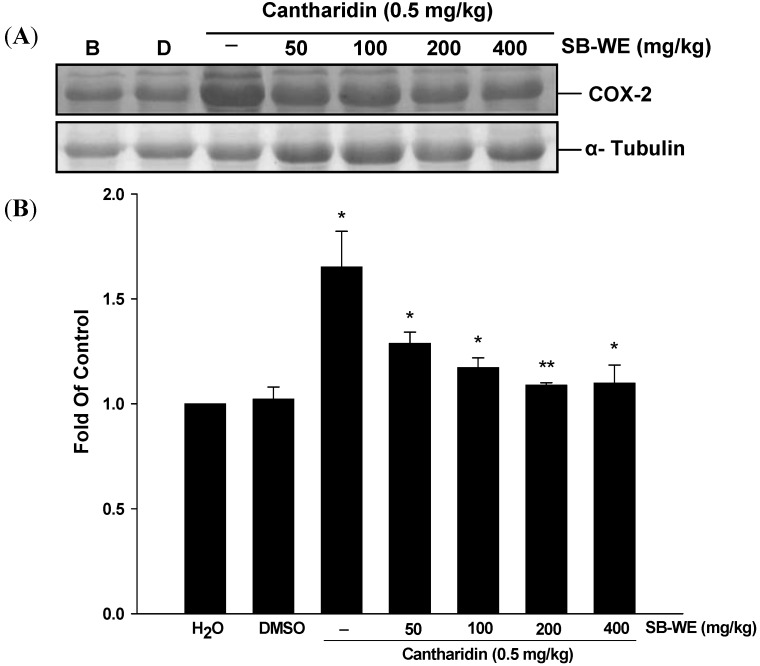
Effects of the boiling-water extract of *S. baicalensis* (SB-WE) against cantharidin-induced rat cystitis and correlated with (**A**)Western blot and (**B**) quantitative analysis (B) of COX-2 protein expression. B, blank; D, DMSO. * *p* < 0.05, ** *p* < 0.001, *n* = 5.

Figure 5 shows that the COX-2 expression level in the cantharidin-treated group was 1.5 fold higher than in the blank group.

### 2.3. Anti-Inflammatory Effects of Boiling-Water Extracts of S. baicalensis (SB-WE)

**Figure 6:**
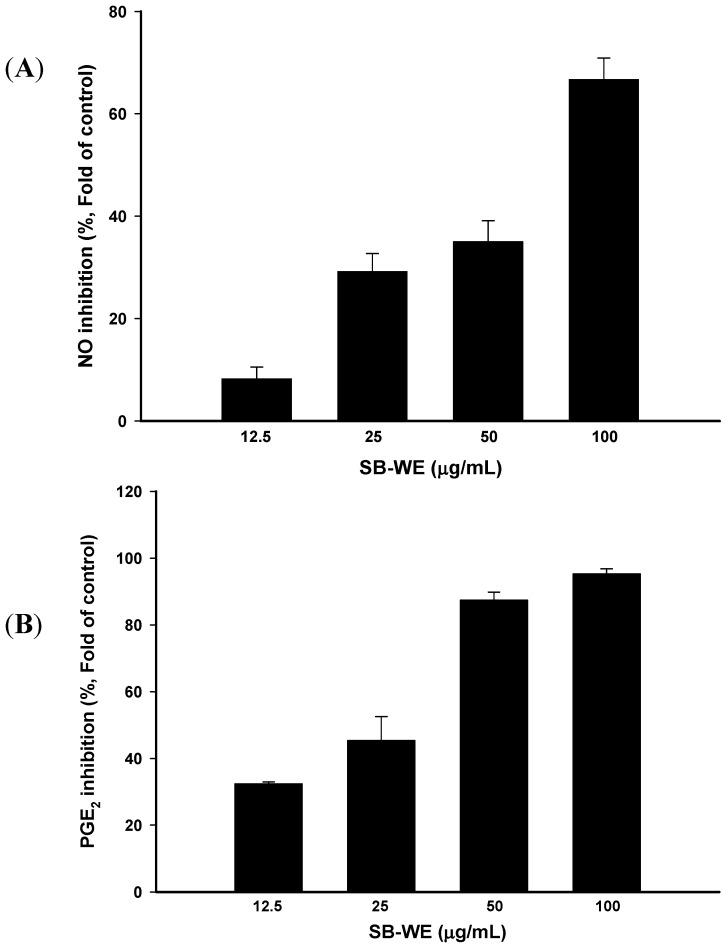
Anti-inflammatory effects of the boiling-water extract of *S. baicalensis* (SB-WE) on (**A**) NO and (**B**) PGE_2_ expressions in LPS-induced RAW 264.7 cells. *n* = 3.

To investigate the anti-inflammatory effects of SB-WE, a LPS-induced RAW 264.7 cell model was used. Results showed that NO and PGE_2_ production by LPS-induced RAW 264.7 cells was significantly inhibited by the SB-WE in a dose-dependent manner, and the respective 50% inhibitory concentration (IC_50_) values were 79.86 and 35.02 μg/mL (Figure 6).

### 2.4. Uroprotective Effects of Boiling-Water Extracts of *S. baicalensis* (SB-WE)

As shown in Figure 3, the SB-WE dose-dependently alleviated the hematuria and increased the urine volume, indicating that the toxicity of cantharidin was reduced by *S. baicalensis*. The hematuria level was reduced from 3+ to 1+ in cantharidin-treated group after oral administration of SB-WE (200 mg/kg). Urine volume in the SB-WE group (200 mg/kg) was about 1.5 fold higher than the cantharidin-treated group (4.0 mL in cantharidin-treated group and 7.5 mL in SB-WE group). In histological analysis, the colliquative necrosis of the urinary bladder epithelium and neutrophil infiltration were attenuated in a dose-dependent manner by administration of SB-WE (Figure 4A). Besides, the signal of c-Fos was also reduced by the SB-WE (Figure 4B, solid black triangles). As shown in Figure 5A, the cantharidin-induced COX-2 overexpression was alleviated by treatment with different dosages of the SB-WE (Figure 5). The above results indicated that cantharidin-induced hematuria and dysuria might be correlated with an inflammatory pathway. The SB-WE displayed significant anti-inflammatory abilities and contributed to prevent cantharidin-induced hematuria in Wistar rats.

### 2.5. Quality Control of the Boiling-Water Extract of *Scutellaria baicalensis* (SB-WE)

**Figure 7:**
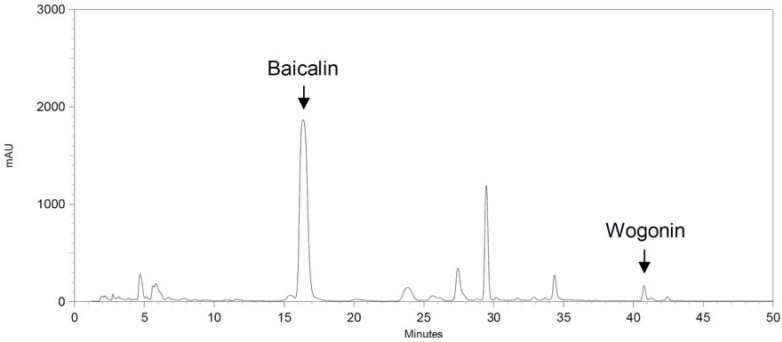
HPLC chromatogram of baicalin and wogonin in the boiling-water extract of *S. baicalensis* (SB-WE). The respective retention times of baicalin and wogonin were 16.5 and 40.9 min.

The HPLC chromatogram profile of the SB-WE is shown in Figure 7. The respective retention times of baicalin and wogonin were 16.5 and 40.9 min. The respective contents of baicalin and wogonin in the SB-WE were 200.95 ± 2.00 and 31.93 ± 0.26 μg/mg.

## 3. Discussion

Cantharidin is a sesquiterpenoid toxicant and can be extracted from many species of blister beetle. In China, mylabris (*Mylabris phalerata* Pallas) is a kind of blister beetle used for anti-cancer or anti-arthritic drugs [21]. However, the therapeutic threshold of mylabris is very narrow. Many adverse reactions of mylabris were reported in clinical use [22,23,24]. According to case reports of cantharidin poisoning, cantharidin induces fever and hematuria in intoxicated patients [25]. However, the mechanisms of cantharidin-induced fever and hematuria are not clear. As shown in Figure 2, cantharidin induced PGE_2_ production and necrosis in T24 cells which were alleviated by NS398, a selective inhibitor of COX-2. Evidence showed that COX-2-dependent cell death was also found in apoptosis-resistant cancer cells treated with an endogenous cannabinoid and anandamide [26]. Hence, we suggest that the possible mechanism of fever and hematuria induced by cantharidin is through induction of COX-2 overexpression by bladder epithelial cells.

In the discovery of uroprotective agents, cyclophosphamide and ifosfamide, two well-known DNA-alkylating agents, are commonly used as hematuria inducers. Metabolites of these two anti-cancer agents, acrolein and 4-hydroxyifosfamide, had been confirmed to be a potential inducers of urothelial irritation. Evidence has shown that hemorrhagic cystitis occurred by directly damaging with these two metabolized products, while lead to the ulceration, edema, neovascularization and necrosis of the bladder [27,28]. Evidence indicates that it takes 12–24 h to induce hemorrhagic cystitis after treatment with cyclophosphamide and ifosfamide [19,20]. In addition, cantharidin could rapidly induce blisters on the skin with topical exposure [29]. Cantharidin is also used as an inflammatory inducer to establish a new animal inflammation model [30], but cantharidin has a high systemic toxicity and causes diarrhea, congestion of the gastrointestinal tract and kidneys *in vivo* [31,32]. In this study, cantharidin was used to induce bladder inflammation and hematuria via direct injection into rat bladder without causing any systemic toxicity within only one day (Figure 3 and Figure 4). After that, the uroprotective effects of *S. baicalensis* were evaluated on the basis of this acute rat hemorrhagic cystitis model. Firstly, results showed that SB-WE inhibited NO and PGE_2_ production by LPS-induced RAW 264.7 cells, indicating that SB-WE displayed strong anti-inflammatory abilities *in vitro* (Figure 6). Secondly, SB-WE also could reduce cantharidin-induced inflammation and hematuria through inhibition of c-Fos and COX-2 expressions in an *in vivo* assay. In TCM, *S. baicalensis* is a medicine widely used as an anti-inflammatory therapy and the characteristic of *S. baicalensis* is to clear internal heat. In addition, *S. baicalensis* is widely used as an anti-pyretic and detoxifying herb and is reported to have anti-cancer, anti-oxidative, and anti-inflammatory effects [14,17,21,33]. A study by Zhang *et al.* showed that *S. baicalensis* could inhibit tumor cell proliferation *via* suppression of COX-2 expression [21]. Our results show that the obvious COX-2 inhibition abilities of *S. baicalensis* contributed to the uroprotective effects against cantharidin-induced hemorrhagic cystitis. Baicalin and wogonin, the principal compounds in root of *S. baicalensis*, display significantly anti-inflammatory effects and have been used as the quality control indicators of the boiling-water extract.

Taken together, we first established a pharmacological animal model for discovering uroprotective agents. The mechanism of cantharidin-induced rat hemorrhagic cystitis was through overexpression of inflammatory-related mediators, c-Fos and COX-2. *S. baicalensis*, a traditional anti-inflammatory herb, displayed significant uroprotective effects against cantharidin-induced hematuria via inhibition of c-Fos and COX-2 overexpression.

## 4. Experimental

### 4.1. Chemicals

Dimethyl sulfoxide (DMSO), 3-[4,5-dimethylthiazol-2-yl]-2,5-diphenyltetrazolium bromide (MTT), *N*-(2-cyclohexyloxy-4-nitrophenyl) methane sulfonamide (NS398) and other chemicals were purchased from Sigma-Aldrich (St. Louis, MO, USA). Dulbecco’s modified Eagle medium (DMEM), fetal bovine serum (FBS), antibiotics, glutamine, and trypsin-EDTA were obtained from Gibco (Grand Island, NY, USA). COX-2 (clone C-20), α-tubulin (clone TU-02), and c-Fos (clone 4) antibodies for Western blotting and immunohistochemistry were purchased from Santa Cruz Biotechnology (Santa Cruz, CA, USA). Acetonitrile and trifluoroacetic acid (TFA) for the HPLC analysis were chromatographic grade and were obtained from Merck (Darmstadt, Germany). Purified deionized water was prepared using the Millipore Milli-RO system (Milli-QRG, Billerica, MA, USA).

### 4.2. Cell Culture

RAW 264.7 (murine macrophage) and T24 (human bladder carcinoma) cell lines were maintained in DMEM, with 10% FBS, 1% L-glutamine, and 1% penicillin-streptomycin, adjusted to contain 3.7 g/L sodium bicarbonate, and were maintained at 37 °C with 5% CO_2_.

### 4.3. Animals

Female Wistar rats (200~250 g) were bought from BioLASCO Taiwan (Yilan County, Taiwan), and maintained at 21 ± 2 °C with food and water *ad libitum*, and kept on a 12-h light/12-h dark cycle. All Wistar rats used in this experiment were cared for according to the Ethical Regulations on Animal Research of Taipei Medical University (approval No.: LAC-98-0050).

### 4.4. Isolation of Cantharidin

Dried mylabris (Chinese blister beetles) was bought from a traditional Chinese medicine store in Taipei, and voucher specimens (MP-002) were deposited in the Graduate Institute of Pharmacognosy Science, Taipei Medical University. The isolation procedure was the same as in our previous study [5]. The structure of cantharidin is shown in Figure 1.

### 4.5. Preparation and Quality Control of the Boiling Water Extract of *S. baicalensis* (SB-WE)

Dried roots of *S. baicalensis* were purchased from a traditional Chinese medicine store in Taipei and identified by Dr. Hsien-Chang Chang. Voucher specimens (SB-001) were deposited in the Graduate Institute of Pharmacognosy Science, Taipei Medical University. The preparation method was modified from our previous study [34]. Radices of *S. baicalensis* were immersed in purified deionized water and boiled for at least 30 min until half of the original amount was left. Aqueous solutions were then filtered, and freeze-dried under vacuum.

Two substance markers, baicalin and wogonin, were used to evaluate the quality of the boiling water extract of *S. baicalensis* (SB-WE). The HPLC equipment comprised an SCL-10Avp System Controller, an SPD-M10A Diode Array Detector, an LC-10ATvp Liquid Chromatograph Pump, an SIL-10Avp Auto Injector, FCV-10Avp Flow-Channel Selection Valves (Shimadzu, Tokyo, Japan), and an ERC-3415 Degasser (ERC, Altegolfsheim, Regensburg, Germany). The stationary phase consisted of a Purospher^®^ STAR RP-18e reversed-phase column (5 μm, 4 mm i.d. × 250 mm, Merck). Acetonitrile-water was used as the mobile phase in the gradient mode as follows: Acetonitrile: 0~17 min, 40%, 17~45 min, 40% to 100%. The flow rate was 1 mL/min, and the oven temperature was maintained at 40 °C. We used a UV wavelength of 280 nm to detect the baicalin and wogonin.

### 4.6. Cytotoxicity, PGE_2_ and NO Inhibitory Assay

The protocols of the cytotoxicity, PGE_2_, and NO inhibitory assays were modified from our previous study [35]. RAW 2624.7 cells (4 × 10^5^ cells/mL) were seeded on 96-well plates and co-treated with LPS (500 ng/mL) and the SB-WE. T24 cells (1 × 10^5^ cells/mL) were seeded on 96-well plates and co-treated with cantharidin and NS398. After 18 h incubation, cytotoxicity was examined by an MTT assay, and the cell culture supernatant was collected for PGE_2_ detection using a commercial assay kit (Assay Designs, Farmingdale, NY, USA). NO was measured as nitrite production in the medium after 24 h of incubation with or without the SB-WE, Briefly, nitrate in the medium was converted to nitrite and measured spectrophotometrically at 540 nm after the Griess reaction.

### 4.7. Cantharidin Induced Hemorrhagic Cystitis in Wistar Rats

Wistar rats were starved for 24 h with free access to water and anesthetized with Zoletil^®^ (Virbac, Carros cedex, France). The urinary bladders of the rats were instilled with cantharidin (0.5 mg/kg) *via* a transurethral injection with a 4-Fr. ureteral catheter (BARD, Murray Hill, NJ, USA). After 1 h, cantharidin-treated rats were orally administrated SB-WE (50, 100, 200, and 400 mg/kg). Each group had five test rats. Urine was continuously collected for 16 h after administration of the SB-WE, and then the rats were sacrificed to obtain the urinary bladders. Hematuria levels were analyzed by a PocketChem^TM^UA analyzer (Arkray, Tokyo, Japan). According to the operation manual, hematuria levels could be divided into four levels: 0+, 1+, 2+ and 3+ depending on the hemoglobulin content in urine. Urinary bladders were fixed in 10% paraformaldehyde, gradually dehydrated in ethanol, and embedded in paraffin. Immunohistochemical staining for c-Fos was performed on formalin-fixed, paraffin-embedded tissue sections. Staining was done using conditions recommended by the vendor. The protocol of COX-2 protein expression was modified from our previous study [5]. COX-2 Protein expression levels were analyzed with the AlphaImager Imaging System.

### 4.8. Statistical Analysis

Data are presented as the mean and standard deviation (SD) and were analyzed with Student’s *t*-test and One-Way ANOVA by SPSS software. The *in vitro* and *in vivo* assays were performed by 3 and 5 independent trials, respectively. The significance level used for statistic analysis displayed as: * *p* < 0.05, ** *p* < 0.001.

## 5. Conclusions

In summary, we found that cantharidin-induced rat hemorrhagic cystitis through up-regulating expression of COX-2 and the inflammation-related transcription factor, c-Fos. In addition, *S. baicalensis* extracts could reduce the cantharidin-induced cytotoxicity, COX-2, PGE_2_ and c-Fos expression *in vitro* and *in vivo*. Our results could be used as a drug screening platform to discover novel uroprotective drugs.
